# Influence of early specialization in world-ranked swimmers and general patterns to success

**DOI:** 10.1371/journal.pone.0218601

**Published:** 2019-06-20

**Authors:** Inmaculada Yustres, Jesús Santos del Cerro, Raúl Martín, Fernando González-Mohíno, Oliver Logan, José María González-Ravé

**Affiliations:** 1 Department of Physical Activity and Sport Sciences, University of Castilla la Mancha, Toledo, Spain; 2 Department of Statistics, University of Castilla la Mancha, San Pedro Mártir, Toledo, Spain; 3 Department of Mathematics, University of Castilla la Mancha, Toledo, Spain; 4 Facultad de Educación y Lenguas, Universidad Nebrija, Madrid, Spain; 5 British Swimming, Sportpark, Loughborough University, Loughborough, United Kingdom; University of Rome, ITALY

## Abstract

**Objectives:**

The primary goal was to examine the influence of early specialization on the performance of senior elite swimmers. Secondly, to provide information about the influence of swim style, distance, sex, status, country, years of high-level competition (YHLC) and age in swimmer’s performance.

**Design:**

Data was obtained from International Federation of Swimming (FINA) regarding the participants 2006–2017 of junior and senior World Championships (WCs). The final filtered database included 4076 swimmers after removing those participating only in junior WCs.

**Method:**

Cramer V coefficient, double and triple-entry tables were used to measure the relationship between the positions occupied in junior and senior phases. A One-Way ANOVA analysis was used to explain the variables time and rank between swimmers who participated in junior and senior WC or just in senior in all the distances and swim styles (SS). A univariate general linear model (GLM) was used to examine the association between time/rank and category (swimmers that participated previously in junior WC or not); YHLC; country; status (highest finishing position: final/semi-final/heats) and age.

**Results:**

Significant differences (p < .001) were found in the GLM, with Rank as dependent variable, for all the variables. Showing that swimmers that participated previously in junior categories obtained greater results in all the interactions, except in 1500m freestyle. Significant differences (p < .001) were found between the variables position and YHLC, showing the variable position improvements as swimmers attended more WCs.

**Conclusion:**

Competing in junior WC has a positive influence in achieve posteriori success in FINA WC. YHLC have a positive impact to achieve better positions.

## Introduction

The influence of the performance obtained in world-class junior categories on the later results in senior categories has been a topic which hasn’t received much research in the sport sciences literature. The vast majority of studies have been focused on the planning and periodization of training used by coaches for elite senior athletes.

On one hand, performance during the initial stages of events has been highlighted as a key component to success in many sports such as bobsledding, skeleton bobsleigh, and swimming [1]. Trying to approach this question, some studies have analyzed different aspects as risks and benefits of youth sport specialization or when athletes reach their peak of performance, [2,3,4,5] and an early age of specialization is increasingly becoming a tendency [6,7,8]. Conversely, other studies showed that early specialization is a large factor in rates of injury and the premature dropout of many talented young athletes [3].

On the other hand, some studies establish that it is common to generalize athlete development as an ascending scale of competition development and this is usually depicted as linear models such as the pyramid analogy [9,10,11]. However, it has also been found that general evidence of linear development from junior to senior was quite scarce (less than 7%) across 256 elite athletes in 27 different sports [8]. Yustres et al. [12] also found that just 17% of finalists of swimming senior WC had previously participated in junior WC.

Although some doubts about the stress tolerance at such a young age have been raised by several scientific organizations such as American Academy of Pediatrics [13,14], high-level competitions are organized at increasingly younger age categories. Since the creation of the junior WC in 2006 this has resulted in a decrease in athletes ages at the time of their international competition debut, leading towards early specialisation and investment in a high amount of training during early stages of development of swimmers [4,5,14,15,16].

This tendency of early specialization generally leads to a greater demand of commitment by youth athletes, creating much more pressure in the physical, psychological and social dimensions [13,17]. Controversial ideas have been discussed as result of this scenario. It has also been debated that it facilitates proper athlete development and allows for career transitions in elite sports such as swimming.

Longitudinal assessment between international competitions and/or seasons can therefore be developed by tracking the swimmers’ performance for a given period of time, analyzing their progression and consistency in performance and help coaches to define realistic goals and to select appropriate training methods [18,19]. This question has been examined by several studies focused on specific samples [12,15,18,20,21], obtaining controversial outcomes. But to date, there is still a lack of knowledge about the influence of performance in junior categories on the results in senior categories focused in elite populations in swimming in a multivariate way.

As it was shown, this study´s landmark investigation draws from junior and senior FINA WCs´ results from 2006 (first junior WC) to 2016. Therefore, taking into account the lack of scientific knowledge about the influence of early specialization in swimmers and their sporty trajectory, this research sought to provide a more detailed, swimming-specific approach to examining the influence of early specialization in elite swimmers. The concept of early specialization is defined in our study as the identification of remarkable performance in important international events at early ages. Secondly, this study sought to provide useful information about the influence of the variables of swim style, distance, sex, status, country, years of high-level competition and age in elite swimmer’s performance showing a general pattern to get top positions in WCs.

## Methods

To assess the relationship between performance in junior WCs and the achievement of success in senior WCs, an observational retrospective study was conducted. Thus, we used historical data from official results websites of the 2007, 2009, 2011, 2013, 2015 and 2016 WCs and 2006, 2008, 2011, 2013, 2015 and 2017 junior WCs.

The data (29928 entries related to 5992 swimmers) underlying the results presented in the study were collected from http://www.fina.org/ and http://www.omegatiming.com/ and processed by the authors. Authors confirm that they did not have any special access privileges and others would be able to access these data in the same manner as the authors. Raw data (29928) was divided first into three differentiated categories: Category 1 (C1), swimmers (3371) who participated in WCs without previous participation in junior WCs; Category 2 (C2), swimmers (1916) who only participated in junior WCs; Category 3 (C3), swimmers (705) who participated in WCs with previous participation in junior WCs. Each entry contains the following explicit variables: Full name, race time, position, status (highest finishing position: final [3], semifinal [2], heats [1]), age, country, gender, distance, swim style, years of high-level competition (the number of years competing at WCs from first participation to last participation) and year of competition. The distances analyzed were 50, 100, 200, 400, 800, 1500 freestyle; 50, 100, 200 backstroke/breaststroke/butterfly and, 200 and 400 individual medley.

The Castilla-La Mancha University Ethical Committee approved this research on November 30^th^ 2016. Informed consent from participants was not necessary because the data was publicly available from the FINA website.

For our purposes, raw data was filtered and sorted, selecting only swimmers from C1 and 3 giving a total of 4076 swimmers from both categories.

The statistical analysis was completed using R software (v. 3.3.3. for Windows). Descriptive statistics were employed for the study to examine the explicative variables. Mean values, ratios and coefficients of dispersion were calculated for the different categories of the target variable, for a better description of basic characteristics of the target population under analysis. Double entry boxes were used to analyze the distribution of the data comparing category with gender, years of high-level competition and position. The information between distances, origin and gender were compared using a triple-entry table. Focusing just on swimmers from C3, a more detailed descriptive analysis was carried out with the aim of investigating if there existed some general patterns to achieve the top positions in senior WCs. Maximum status in junior and senior WCs was selected for each swimmer.

The relationship between the positions achieved in junior and subsequently in senior phase has been studied within C3 swimmers. Confirming, from a descriptive point of view, that correlation exists between the positions occupied in junior and lately in senior. For this purpose, the Cramer V coefficient is used to measure the intensity of the association between qualitative variables. The detailed analysis of the double-entry tables will allow us to study the meaning of the association.

Furthermore, in order to study more deeply the existence of statistically significant differences between swimmers that have participated in junior and senior WCs, we will apply a One-Way ANOVA analysis. The variables examined will be both mean Time and Rank between swimmers that participated only in senior WCs (C1) and those swimmers who prior participated in junior WCs (C3) in all the distances and swim styles. This will allow us to identify whether or not there are significant differences between the two types of swimmers and to measure, if applicable, the size of these differences. The criterion for statistical significance was set at an alpha level of 0.05.

Finally, a univariate general linear model (GLM) was used to examine the association between the dependent variables time or rank and the independent variables origin (previous participation in junior category or not), years of high-level competition, gender and age in all the distances and swim styles. A general model was carried out with Rank as dependent variable and the variables origin, years of high-level competition, age and gender as independent. Coefficients R^2^ have been calculated as well as global significance test for the estimation of these models. This was to investigate if the variables included in the models significantly influence with the time or rank. Each of the explanatory variables were evaluated one by one, and it has generally been shown to be significant. Setting p-value less than 5% in the lineal regression model as significant. Model assumptions of normality, homoscedasticity and independence were tested using the Kolmogorov-Smirnov test, Levene’s test for homoscedasticity and the test for independence, respectively. All the residuals showed a satisfactory pattern.

## Results

Of 4076 swimmers analyzed in our study, 3371 (82.70%) participated directly in WCs (C1), with only 705 (17.29%) participating in both championships (C3). Russia (RUS), Italy (ITA) and Spain (ESP) were the countries that had the most swimmers from C3 (45.20%, 40.11% and 37.50% respectively) (Table 1). A total of 47.86% and 44.98% were female and 52.14% and 55.02% male in C1 and C3, respectively.

**Table 1:** Number of swimmers for the interactions status*category*country (%).

COUNTRY	ORIGIN		STATUS		
			HEATS	SEMIFINAL	FINAL
**TOTAL** **N = 4076**	**C1**	N = 3371 (82.70%)	78.33%	12.80%	8.87%
	**C3**	N = 705 (17.29%)	76–40%	12.96%	10.64%
**USA** **N = 245**	**C1**	N = 181 (73.88%)	13.81%	1215%	74.03%
	**C3**	N = 64 (26.12%)	27.78%	16.67%	55.56%
**RUS** **N = 177**	**C1**	N = 97 (54.80%)	40.21%	24.74%	35.05%
	**C3**	N = 80 (45.20%)	25.00%	32.69%	42.31%
**AUS** **N = 212**	**C1**	N = 164 (77.36%)	23.17%	27.44%	49.39%
	**C3**	N = 48 (22.64%)	32.14%	32.14%	35.71%
**CHN** **N = 280**	**C1**	N = 230 (82.14%)	57.83%	13.91%	28.26%
	**C3**	N = 50 (17.86%)	54.55%	21.21%	24.24%
**JPN** **N = 187**	**C1**	N = 148 (79.14%)	36.49%	29.73%	33.78%
	**C3**	N = 39 (20.86%)	23.08%	38.46%	38.46%
**CAN** **N = 178**	**C1**	N = 112 (62.92%)	51.79%	19.64%	28.57%
	**C3**	N = 66 (37.08%)	50.00%	21.05%	28.95%
**HUN** **N = 93**	**C1**	N = 83 (89.25%)	33.73%	21.69%	44.58%
	**C3**	N = 10 (10.75%)	60.00%	20.00%	20.00%
**GBR** **N = 175**	**C1**	N = 83 (89.25%)	28.67%	26.57%	44.76%
	**C3**	N = 10 (10.75%)	36.36%	27.27%	36.36%
**ESP** **N = 72**	**C1**	N = 45 (62.50%)	42.22%	31.11%	26.67%
	**C3**	N = 27 (37.50%)	42.86%	9.52%	47.62%
**ITA** **N = 182**	**C1**	N = 109 (59.89%)	48.62%	24.77%	26.61%
	**C3**	N = 73 (40.11%)	45.10%	21.57%	33.33%

USA: United States of America; RUS Russia; CHN: China; AUS: Australia; JPN: Japan; HGR: Hungary; ESP: Spain; CAN: Canada; GBR: Great Britain; ITA: Italy

Additionally, the proportion of C3 data which reached the final (status 3), was slightly higher (10.64%) than in C1 (8.87%). There was similarity in semifinalist positions (status 2) (12.96% and 12.80%) and higher in heats (status 1) for C1 (78.33% and 76.40% respectively). The most representative 10 countries were included in the analysis in order to measure the percentage of participation in each status. (Table 1).

The analysis was repeated, including the variable gender instead of country. The percentage of swimmers who participated in senior WCs final from C3 was 20.17% for women and 15.14% for men; semifinal 14.01% and 10.20%; heats 65.81% and 74.66% (women and men respectively). Moreover, the percentage of swimmers that participated in senior WCs final from C1 was 19.79% and 14.51%; semifinal 15.19% and 12.01%; heats 65.02% and 73.48% (women and men respectively).

Focusing just on swimmers from C3, a more detailed descriptive analysis was carried out with the aim of investigating if there existed some general trajectory patterns to achieve the top positions in senior WCs. Maximum status in junior and senior WCs was selected for each swimmer. Results from the descriptive analysis of C3 swimmers showed that 39.68% of finalists in senior WC had previously participated as finalists in the junior category. An interesting result was that 92.71% of swimmers that had their highest finishing position as the heats (swimmers that did not classify to semifinal) in senior, had also previously failed in reaching the semifinal and final in junior WCs. The same analysis was performed for USA, RUS, AUS and CHN, due to the large percentage of swimmer participations. USA showed that 66.67% of swimmers that participated in heats and final in junior WCs later in their careers reached the final in senior WCs. Also, RUS and AUS’ swimmers (45% and 53.85% respectively) that reached top positions in junior also reached the final in the senior category. 80% of Chinese semifinalist swimmers in junior category had their highest finishing position as heats in senior WCs. (Table 1).

One-Way ANOVA focusing on the performance measurement was carried out. Where significant differences (p < .01) were found in some of the interactions (Table 2) when comparing mean Time and Rank between swimmers who participated directly in senior WCs (C1) and those swimmers who prior participated in junior WCs (C3) in all the distances and swim styles.

**Table 2:** ANOVA. category*time rank*distance*swimstyle.

TIME	SWIM STYLE									
	1		2		3		4		5	
	C1	C3	C1	C3	C1	C3	C1	C3	C1	C3
50m	26.13	24.76*	28.64	28.00*	31.47*	30.30	27.01	26.34*	-	-
	Df (1;1856)		Df (1;1209)		Df (1;1252)		Df (1;1445)		-	
	F = 33,43		F = 6.624		F = 10.81		F = 7.908			
100m	55.11	53.81*	60.93	60.11	67.43	65.99*	57.54	57.77	60.21	57.92*
	Df (1;1739)		Df (1;1266)		Df (1;1168)		Df (1;1252)		Df (1;189)	
	F = 13.18		F = 3.141		F = 5.306		F = 0.333		F = 5.472	
200m	117.81	117.17	128.12	127.55	142.57	140.07*	125.08	125.52	129.67	130.25
	Df (1;1291)		Df (1;792)		Df (1;896)		Df (1;789)		Df (1;866)	
	F = 0.787		F = 0.458		F = 5.578		F = 0.317		F = 0.396	
400m	247.08	246.16	-	-	-	-	-	-	272.95	274.00
	Df (1;798)		-		-		-		Df (1;565)	
	F = 0.316								F = 0.295	
800m	505.69*	513.19	-	-	-	-	-	-	-	-
	Df (1;548)		-		-		-		-	
	F = 4.874									
1500m	946.78	948.30	-	-	-	-	-	-	-	-
	Df (1;452)		-		-		-		-	
	F = 0.039									
RANK	SWIM STYLE									
	1		2		3		4		5	
	C1	C3	C1	C3	C1	C3	C1	C3	C1	C3
50m	63.05	43.68*	36.98	30.24*	38.70	36.02	49.25	40.50*	-	-
	Df (1;1856)		Df (1;1209)		Df (1;1252)		Df (1;1445)		-	
	F = 39.87		F = 8.391		F = 0.923		F = 8.173			
100m	57.82	51.52*	34.57	31.48	36.06	36.65	37.00	36.89	31.38	23.96
	Df (1; 1739)		Df (1;1266)		Df (1;1168)		Df (1;1252)		Df (1;189)	
	F = 4.769		F = 2.573		F = 0.054		F = 0.002		F = 3.757	
200m	38.51	36.11	20.83	21.15	22.27	22.61	18.95	20.90	23.12	24.98
	Df (1;1291)		Df (1;792)		Df (1;896)		Df (1;789)		Df (1;866)	
	F = 1.147		F = 0.049		F = 0.05		F = 1.877		F = 1.193	
400m	28.55	29.37	-	-	-	-	-	-	17.74*	20.29
	Df (1;798)		-		-		-		Df (1;565)	
	F = 0.189								F = 3.369	
800m	18.53*	22.14	-	-	-	-	-	-	-	-
	Df (1;548)		-		-		-		-	
	F = 6.299									
1500m	14.64*	19.82	-	-	-	-	-	-	-	-
	Df (1;452)		-		-		-		-	
	F = 15.18									

Df, Degrees of freedom; F, F statistic.

*Significant differences (p < .01) with better results for that category; -, No data available, small size for carrying out the analysis.

A general model was carried out with Rank as dependent variable and the variables origin, years of high-level competition, age and gender as independent. Significant differences (p < .001) were found for all the variables in the general model for the variable Rank. Firstly, showing that swimmers from C3 reached better finishing position than swimmers from C1, (mean Rank 32.91 and 37.92 respectively).

The model confirms a strong association between the variables position obtained and years of high-level competition, YHLC. Showing that, each subsequent WCs that swimmers compete in, they get 3.02 higher ranking positions. Better positions were evidenced for swimmers as they got one year older and women were better than men (3.40 and 14.59 respectively). (Table 3).

**Table 3:** General model. Rank variable.

	Estimate	Std.Error	t value	Pr(>|t|)
**(Intercept)**	109.42286	1.40945	77.64	<2e-16 ***
**C3**	-9.01638	0.70194	-12.85	<2e-16 ***
**YHLC**	-3.02247	0.25371	-11.91	<2e-16***
**Age**	-3.40379	0.07127	-47.76	<2e-16***
**Woman**	14.59583	0.48620	30.02	<2e-16***

YHLC, Years of high-level competition.

Signif. codes: 0 ‘***’

When including the variables distance and swim style to the model, a large percentage of their interactions with the variables showed significant differences (p < .01). The variable origin showed significant differences (p < .01) where swimmers from C3 reached better position than swimmers from C1 in all of them, except in 1500m freestyle where swimmers from C1 achieved 5.82 positions higher. Also, significant differences (p < .01) where found with gender and age. Better positions are obtained by women compared to men and also by increasing one year of age for all the distance and swim styles.

For the variable years of high-level competition, all interactions showed significant differences (p < .01) except 100 individual medley, 200 breaststroke, 400 freestyle and individual medley. (Table 4)

**Table 4:** Distance and swim style interactions. Better performance for C3, YHLC, YO, M. Significant ranking raise for C3, YHLC, YO, W.

**BETTER PERFORMANCE**		**Freestyle**	**Backstroke**	**Breaststroke**	**Butterfly**	**Individual Medley**
**50 m**	C3	-1.45***	-1.04***	-1.64***	-1.33***	-
	YHLC	-0.47***	-0.50***	-0.41***	-0.34***	-
	YO	-0.23***	-0.25***	-0.35***	-0.26***	-
	M	-2.89***	-2.67***	-3.22***	-2.68***	-
		R^2^ 0.31	R^2^ 0.39	R^2^ 0.34	R^2^ 0.36	-
		Df(4;1853)	Df(4;1206)	Df(4;1249)	Df(4;1442)	
		F = 208.4	F = 190.8	F = 159.1	F = 202	
**100 m**	C3	-1.91***	-2.18***	-2.12***	-1.01**	-2.52***
	YHLC	-0.30**	-0.55**	-0.76***	-0.45***	-
	YO	-0.49***	-0.66***	-0.61***	-0.44***	-0.53***
	M	-5.08***	-4.67***	-6.31***	-5.64***	-6.18***
		R^2^ 0.39	R^2^ 0.34	R^2^ 0.36	R^2^ 0.47	R^2^ 0.48
		Df(4;1736)	Df(4;1263)	Df(4;1165)	Df(4;1249)	Df(4;186)
		F = 272.9	F = 163.3	F = 161.8	F = 274.4	F = 43.78
**200 m**	C3	-2.22***	-2.40**	-2.54**	-	-
	YHLC	-0.66**	-0.79**	-0.88*	-0.49*	-1.24***
	YO	-1.01***	-0.96***	-1.06***	-0.57***	-0.80***
	M	-9.83***	-10.91***	-14.10***	-11.42***	-11.57***
		R^2^ 0.42	R^2^ 0.47	R^2^ 0.47	R^2^ 0.53	R^2^ 0.46
		Df(4;1288)	Df(4;789)	Df(4;893)	Df(4;786)	Df(4;863)
		F = 235.8	F = 177.6	F = 203.4	F = 219.8	F = 182.6
**400 m**	C3	-6.23***	-	-	-	-
	YHLC	-	-	-	-	-1.89**
	YO	-2.40***	-	-	-	-1.36***
	M	-18.37***	-	-	-	-24.45***
		R^2^ 0.43	-	-	-	R^2^ 0.53
		Df(4;795)				Df(4;562)
		F = 149.2				F = 158.2
**800 m**	C3	-	-	-	-	-
	YHLC	-	-	-	-	-
	YO	-3.36***	-	-	-	-
	M	-35.27***	-	-	-	-
		R^2^ 0.50				
		Df(4;545)				
		F = 137.5				
**1500 m**	C3		-	-	-	-
	YHLC	-6.93***	-	-	-	-
	YO	-3.04***	-	-	-	-
	M	-64.02***	-	-	-	-
		Df(4;449)	-	-	-	-
		F = 90.32				
**RANKING RAISE**		**Freestyle**	**Backstroke**	**Breaststroke**	**Butterfly**	**Individual Medley**
**50 m**	C3	-22.19***	-8.49***	-7.86**	-10.96***	-
	YHLC	-6.41***	-4.21***	-3.80***	-6.09***	-
	YO	-4.55***	-3.26***	-3.53***	-3.79***	-
	M	-19.19***	-5.28**	-21.02***	-23.17***	-
		R^2^ 0.27	R^2^ 0.27	R^2^ 0.26	R^2^ 0.26	-
		Df(4;1853)	Df(4;1206)	Df(4;1249)	Df(4;1442)	
		F = 171.4	F = 112.4	F = 108.7	F = 129.6	
**100 m**	C3	-14.79***	-5.92**	-5.14*	-	-10.28**
	YHLC	-2.17*	-2.81***	-3.07***	-4.13***	-
	YO	-5.58***	-3.45***	-3.21***	-3.16***	-2.12***
	M	-24.29***	-8.86***	-20.76***	-18.81***	-12.69***
		R^2^ 0.28	R^2^ 0.26	R^2^ 0.23	R^2^ 0.25	R^2^ 0.24
		Df(4;1736)	Df(4;1263)	Df(4;1165)	Df(4;1249)	Df(4;186)
		F = 170.2	F = 112.4	F = 89.24	F = 103.6	F = 14.88
**200 m**	C3	-7.48***	-	-4.35**	-	-
	YHLC	-2.38**	-1.28*	-	-1.60**	-2.21***
	YO	-4.06***	-1.93***	-2.07***	-1.21***	-2.18***
	M	-13.52***	-4.90***	-7.90***	-6.92***	-10.43***
		R^2^ 0.26	R^2^ 0.18	R^2^ 0.17	R^2^ 0.14	R^2^ 0.22
		Df(4;1288)	Df(4;789)	Df(4;893)	Df(4;786)	Df(4;863)
		F = 111.1	F = 42.09	F = 46.13	F = 31.57	F = 61.75
**400 m**	C3	-	-	-	-	-
	YHLC	-	-	-	-	-
	YO	-2.91***	-	-	-	-1.28***
	M	-11.96***	-	-	-	-5.32***
		R^2^ 0.25				R^2^ 0.16
		Df(4;795)				Df(4;562)
		F = 67.87				F = 26.94
**800 m**	C3	-	-	-	-	-
	YHLC	-1.18*	-	-	-	-
	YO	-1.48***	-	-	-	-
	M	-3.67***	-	-	-	-
		R^2^ 0.19				
		Df(4;545)				
		F = 32.42				
**1500 m**	C3	-5,82***	-	-	-	-
	YHLC	-2.26***	-	-	-	-
	YO	-0.60***	-	-	-	-
	M	-6.25***	-	-	-	-
		R^2^ 0.21				
		Df(4;449)				
		F = 29.04				

Df, Degrees of freedom; F, F statistic; C3, category 3; YHLC, each more year of high-level competition; YO, each year old; W, women; M, men.

Signif. codes: 0

‘***’ 0.001

‘**’ 0.01

‘*’ 0.05; -, No data available, small size for carrying out the analysis.

On the other hand, focusing on the variable Time and its interactions with the variables origin, years of high-level competition, age and gender in each of the distances and swim styles, several models were created. These models’ results showed significant differences (p < .01) with most of the variables as shown in the table below (Table 4). Swimmers from C3 achieved significantly (p < .01) better results than swimmer from C1 in all the interactions except in 200 butterfly, 200 individual medley, 400 individual medley, 800 and 1500 freestyle, where results were not significant. Conversely, swimmers from C1, were 5.15 seconds faster than swimmers from C3 (p < .01) in 1500m freestyle.

As the athletes’ years of high-level competition increases, times are significantly (p < .01) better, except for 100 individual medley, 400 and 800 freestyle.

Significantly (p < .01) lower times were achieved for men compared to women in all the distances and swim styles. (Table 4)

## Discussion

The primary purpose of this research was to provide a more detailed, swimming-specific approach to examining the influence of early specialization in world elite swimmers taking into account all swimmers finishing position in FINA World Championships from 2007 to 2016. The second purpose was, to provide useful information about the influence of the variables swim style, distance, sex, status, country, years of high-level competition and age in elite swimmer’s performance showing a general pattern to achieve top positions in WCs. We have compared the performance in FINA World Championships between the swimmers that competed previously in junior squads and the swimmers that went straight to the senior squads. Significant differences (p < .01) were found between C1 and C3 swimmers for mean Time and Rank in several distances and swim styles. Opposite findings to the study of Yustres et al. [12] where no significant differences (p < .01) were found for Rank and Time when analyzed differences between C1 and C3 swimmers but just focused on the finalists in a previous study.

Although, according to some other studies [12,22,23] the ratio of conversion (17.29%) from junior to senior was low, our results showed that swimmers that had previously participated in junior WCs reached 9.01 better positions than C1 swimmers in senior WCs. Therefore, although noticing the low ratio of conversion from junior to senior category, we do find evidence to suggest that for swimmer who progress from one category to the next, early specialization does become a significant factor to achieve better results in senior categories. Moreover, 39.68% of those swimmers who evolve from one category to the next, reached the final in senior WC.

These results are aligned with the study of Svendsen et al. [24] where race performance at junior level was found to be a strong predictor of later success as a senior elite cyclist. Also, Neeru et al. [16] affirmed that some degree of sports specialization is necessary to develop elite-level skill development. Early sports specialization has been affirmed to be necessary for skill acquisition required for competitive success in many sports [25]. In swimming, considered an early specialization sport, extensive training must be performed from an early age in order to achieve long-term high performance. [26]

Additionally, our model confirmed that positions and marks were significantly (p < .01) better when increasing the years of high-level competition, achieving 3.02 higher finishing positions every WCs. These results are in line with Yustres et al. [12] who affirmed that there is a strong association between the position obtained and the years at high level competition, getting better positions as they attend more WCs. In this sense,more importance is even granted to the early specialization factor. Not only being a crucial factor as has been expressed before, but also as it allows swimmers to participate in a greater number of WCs, thus improving the positions obtained at the senior level.

It has been affirmed that early emphasis on obtaining good results is associated with major rates of depletion and dropout when the athletes might be exposed to higher levels of pressure and stress [27]. However, Lang & Light [28] affirmed early success not to be the main cause of later failures and drop-outs. Also, Yustres et al. [12] affirmed that there are many more influential factors (family and economic support, injuries, etc) that affect motivation and the desire to participate that restrict the longevity of successful young swimmers. Therefore, bearing in mind that early specialization has been shown to be a crucial factor and as more years of high-level competition better are the results in senior category, from a long-term athlete development perspective, it is interesting to examine aside from early predictors of success, which factors might be associated with dropout [24] and which ones are worth taking into account for young athletes to achieve successful careers.

Trying to show general patterns of performance development in swimming, it is worth noting that linear trajectories and swimmers’ performance stability are quite difficult to maintain at a high level. There is a rather long list of research studies measuring swimmers’ background and trajectory models in one way or another [15,18,19,20]. However, none of them provide the information about the specific factors than can lead to success in world-class Competitions. Then, aiming to show these patterns of performance for elite swimmers, several variables were analysed in our model. When including the variables distance and swim style, 1500m freestyle was the only event which showed that participating in junior WCs prior to senior WCs was not correlated to achieve remarkable results. This fact could be explained by the results of Allen et al. [29] who found that the peak of performance occurred at later ages for the shorter distances for both sexes (~1.5–2.0 years between sprint and distance-event groups). Therefore, long distance swimmers may reach their best performance in junior category, failing in maintaining the demanding performance in senior category. The variable age confirmed significant performance enhancement, as swimmers’ get older. Results that may be related to the variable years of high-level competition, since the swimmers get older, they are more likely to participate in world championships. Allen et al. [29], found that medallists showed slightly higher age of peak performance compared with non-medallists for men and women.

In this sense, according to Costa et al. [18] coaches should have a long-term view when considering training design and periodization of world-ranked swimmers. Also, it has been concluded that large talent-development squads are required to include most eventual Olympic qualifiers [29]. Controversial results were found where the variable age was not statistically significant (*p >* .05) when comparing the times obtained in senior WCs between swimmers with or without previous participation in junior WCs. [12]

When taking into account the variable country, RUS, ITA and ESP were the countries which showed the highest ratio of conversion from junior to senior (45.20%, 40.11% and 37.50% respectively). Moreover, RUS and AUS showed that swimmers (45% and 53,85% respectively) who got top positions in junior also reached the final in senior. Only 26,12% of American swimmers participated in junior WCs prior senior WCs. This result is in line with Sokolovas et al. [21] who affirmed that most of the American elite level swimmers at the age of 18 were unknown in the Top-100 at younger ages. However, although bearing in mind this scarce progress from one category to the next, 66.67% of USA swimmers who progressed racing in final in junior WCs later reached the final in senior WCs. Highlighting the importance not only of participating in junior WCs but also of reaching good positions. The variable gender showed that C3 women (20.17%) showed the maximum number of participations in final and semi-final.

Nevertheless, there are several factors that can also play a major role in succeeding. Better coaching, together with better support, allows swimmers to have better training conditions, and might in some cases, contribute to a higher professional environment to achieve better performances [18]. There are some other factors affecting swimmer’s career and should be considered for future studies.

## Conclusions

We conclude that participation in junior WCs have positive influence in achieving success in FINA WCs.

Years of high-level competition have a positive impact to achieve better positions and marks. In all the distances and swim styles men achieve lower marks than women. Coaches might consider early specialization as a means to achieve success in the highest levels of competition [24, 26]. Taking into account the stability of swimmer’s performance, federations need to invest in strategies to increase the conversion ratio as competing in junior category and experience counts [12].

## Practical implications

- Guide to coaches and swimmers about general patterns to follow to success in international competitions. Developing documents with data from elite swimmers that reached the final in senior WCs as a model for their career.
- Useful information to federations, High Performance Centers and national teams about characterization of elite junior swimmers. Then, they could use this information for talent detection programs.
- Statistical evidence to the real-world setting of sport and exercise about the influence of early specialization as well as its risks or limitations.

## References

[1] West DJ, Owen NJ, Cunningham DJ, Cook CJ, Kilduff LP. Strength and power predictors of swimming starts in international sprint swimmers. J Strength Cond Res. 2011;25(4): 950–955. 10.1519/JSC.0b013e3181c8656f 20664366

[2] Capranica L, Millard-Stafford M. Youth Sport Specialization: How to Manage Competition and Training? Int J Sports Physiol Perform. 2011;6: 572–579. 22174125 10.1123/ijspp.6.4.572

[3] Fabricant P, Lakomkin N, Sugimoto D, Tepolt FA, Stracciolini A, Kocher MS. Youth sports specialization and musculoskeletal injury: a systematic review of the literature. Phys Sports med. 2016 9;44(3): 257–62. 10.1080/00913847.2016.1177476 27121730

[4] Baker J. Early specialization in youth sport: a requirement for adult expertise?. High Abil Stud. 2003;14: 85–94.

[5] Wiersma L. Risks and benefits of youth sport specialization. Perspectives and recommendations. Pediatr Exerc Sci. 2000;12: 13–22.

[6] Côté J. The influence of the family in the development of talent in sport. Sport Psychol. 1999;13: 395–417.

[7] Hill G. Youth sport participation of professional baseball players. Sociol Sport J. 1993;10: 107–114.

[8] Gulbin J, Weissensteiner J, Oldenziel K, Gagné F. Patterns of performance development in elite athletes. Eur J Sport Sci. 2013;13(6): 605–14. 10.1080/17461391.2012.756542 24251738

[9] Bailey RP, Collins D, Ford PA, MacNamara A, Toms M, Pearce G. Participant development in sport: An academic literature review. 2010

[10] Green B. Building sport programs to optimize athlete recruitment, retention and transition: Toward a normative theory of sport development. J Sport Manage. 2015;19: 233–253.

[11] Stewart B, Nicholson M, Smith A, Westerbeek H. Australian sport: Better by design? The evolution of Australian sport policy. London and New York: Routledge; 2004. ISBN 0415340462

[12] Yustres I, Martín R, Fernández L, González-Ravé JM. Swimming championship finalist positions on success in international swimming competitions. PLoS ONE 2017;12(11) 10.1371/journal.pone.0187462 PMC5673220 29108018

[13] Intensive training and sport specialization in young athletes. American Academy of Pediatrics. Pediatrics. 2000;106: 154–157 10878168

[14] Pizzuto F, Bonato M, Vernillo G, La Torre A, Piacentini MF. Are the World Junior Championship Finalists for Middle and Long-Distance Events Currently Competing at International Level?. IJSPP. 2016;12(3) 10.1123/ijspp.2015-0717 27294320

[15] Allen S, Vandenbogaerde T, Pyne D, Hopkins W. Predicting a nation´s Olympic-qualifying swimmers. Int J Sports Physiol Perform. 2015;10: 431–435 10.1123/ijspp.2014-0314 25365394

[16] Neeru J, Dugas L, LaBella C. Sports Specialization in Young Athletes: Evidence-Based Recommendations. Sports Health. 2013;5 10.1177/1941738112464626 PMC3658407 24427397

[17] Baker J, Cobley S, Fraser-Thomas J. What do we know about early sport specialization? Not much! HIGH ABIL STUD. 2009;20(1): 77–89

[18] Costa M, Marinho D, Reis V, Silva A, Marques M, Bragada J, et al Tracking the performance of world-ranked swimmers. J Sports Sci Med. 2010;9: 411–417 24149635 PMC3761712

[19] Pyne D. Progression and variability of competitive performance of Olympic swimmers. J Sports Sci. 2014;22: 613–620 10.1080/02640410310001655822 15370491

[20] Costa M, Marinho D, Bragada J, Silva A, Barbosa T. Stability of elite freestyle performance from childhood to adulthood. J Sports Sci. 2011; 1–7 10.1080/02640414.2011.587196 21777055

[21] Sokolovas G, Vilas-Boas JP, Alves F, Marques A. Analysis of USA swimming’s all-time top 100 times. In: X International Symposium on Biomecanics and Medicine in Swimming. Rev Port Cien Desp. 2006; 315–317

[22] Durand-Bush N, Salmela J. The development and maintenance of expert Athletic performance: perceptions of World and Olympic champions. J Appl Sport Psychol. 2002;14: 154–171.

[23] Barreiros A, Côté J, Fonseca AM. From early to adult sport success: Analysing athletes' progression in national squads. Eur J Sport Sci. 2002;14: 178–182. 10.1080/17461391.2012.671368 24444203

[24] Svendsen IS, Tønnesen E, Tjelta LI, Orn S. Training, Performance and Physiological Predictors of a Successful Elite Senior Career in Junior Competitive Road Cyclists. Int J Sports Physiol Perform. 2018;10: 1–20 10.1123/ijspp.2017-0824 29745739

[25] Brenner JS; AAP COUNCIL ON SPORTS MEDICINE AND FITNESS. Sports Specialization and Intensive Training in Young Athletes. Pediatrics. 2016;138(3): e20162148 10.1542/peds.2016-2148 27573090

[26] Balyi I, Way R, Higgs C. Long-term athlete development United States of America: Sheridan Books; 2013.

[27] Gould D, Tuffey S, Udry E, Loehr J. Burnout in competitive junior tennis players: II. Qualitative analysis. Sport Psychol. 1996;10: 341–366.

[28] Lang M, Light R. Interpreting and implementing the long-term athlete development model: English swimming coaches' views on the (swimming) LTAD in practice. Int J Sports Sci Coach. 2010;5(3): 389–402.

[29] Allen S, Vandenbogaerde T, Hopkins W. Career performance trajectories of Olympic swimmers: benchmarks for talent development. Eur J Sports Sci. 2014;14(7): 643–641 10.1080/17461391.2014.893020 24597644

